# Peramivir and laninamivir susceptibility of circulating influenza A and B viruses

**DOI:** 10.1111/irv.12187

**Published:** 2013-11-07

**Authors:** Sook-Kwan Leang, Simon Kwok, Sheena G Sullivan, Sebastian Maurer-Stroh, Anne Kelso, Ian G Barr, Aeron C Hurt

**Affiliations:** a WHO Collaborating Centre for Reference and Research on InfluenzaNorth Melbourne, Vic., Australia; b Bioinformatics Institute (BII), Agency for Science, Technology and Research (A*STAR)Singapore City, Singapore; c School of Biological Sciences (SBS), Nanyang Technological University (NTU)Singapore City, Singapore; d National Public Health Laboratory (NPHL), Communicable Diseases Division, Ministry of Health (MOH)Singapore City, Singapore; e School of Applied Sciences and Engineering, Monash UniversityChurchill, Vic., Australia

**Keywords:** Antiviral susceptibility, laninamivir, neuraminidase inhibitors, peramivir

## Abstract

Influenza viruses collected from regions of Asia, Africa and Oceania between 2009 and 2012 were tested for their susceptibility to two new neuraminidase inhibitors, peramivir and laninamivir. All viruses tested had normal laninamivir inhibition. However, 3·2% (19/599) of A(H1N1)pdm09 viruses had highly reduced peramivir inhibition (due to H275Y NA mutation) and <1% (6/1238) of influenza B viruses had reduced or highly reduced peramivir inhibition, with single occurrence of variants containing I221T, A245T, K360E, A395E, D432G and a combined G145R+Y142H mutation. These data demonstrate that despite an increase in H275Y variants in 2011, there was no marked change in the frequency of peramivir- or laninamivir-resistant variants following the market release of the drugs in Japan in 2010.

## Introduction

Currently, the neuraminidase inhibitors (NAIs) are the only class of antivirals that are effective against circulating influenza A and B viruses due to widespread resistance against the older adamantane class of drugs.^1^ Two NAIs, orally administered oseltamivir (Tamiflu®) and inhaled zanamivir (Relenza®), have been licensed in many countries since 1999. In 2010, two new NAIs, peramivir (Rapiacta®) and laninamivir (Inavir®), were licensed in Japan.^2^ Peramivir has also been licensed in South Korea^3^ and most recently in China, as a response to concerns of A(H7N9) avian influenza.^4^ Peramivir is delivered intravenously and therefore is well suited to treating severely ill patients who may be unable to use oral or inhaled formulations.^5^ Laninamivir octanoate, a prodrug of laninamivir, is administered by inhalation (in a powdered form) and is quickly converted into its active form in the lungs.^6^ However, compared with oseltamivir and zanamivir, which are administered twice daily for 5 days, laninamivir is long-acting, requiring only a single administration for retention of the drug for at least five days.^7^ During 2011–2012, laninamivir was the largest selling NA inhibitor in Japan.^8^ As with any new antiviral agents that are released to the market, it is important to monitor circulating influenza strains for the development of resistance to both peramivir and laninamivir.^9^–^11^ This is particularly pertinent given the widespread resistance to the NAI oseltamivir that occurred in seasonal A(H1N1) viruses in 2008.^12^,^13^ Here, we evaluate the peramivir and laninamivir susceptibility of influenza A and B viruses circulating in parts of Asia, Africa and Oceania between 2009 and 2012, a time frame that spans pre- and post-market launch of these drugs in Japan and South Korea.

## Sample selection and classification criteria

Approximately 2500 influenza A and 1238 influenza B viruses collected from 19 countries and territories in Asia, Africa and Oceania via the WHO Global Influenza Surveillance and Response System (GISRS) between 2009 and 2012, were screened for peramivir and laninamivir susceptibility. Viruses were received from the following countries and territories: Australia (2492), Brunei (5), Cambodia (164), Fiji (23), Hong Kong (4), Kenya (8), Macau (SAR, China) (101), Malaysia (44), New Caledonia (9), New Zealand (457), Papua New Guinea (10), Philippines (120), Singapore (196), Solomon Islands (1), South Africa (17), South Korea (10), Sri Lanka (27), Taiwan (6) and Thailand (92). Peramivir susceptibility and laninamivir susceptibility were assessed using a functional fluorescence-based NA inhibition assay as described by Hurt *et al*.^14^ Sequence analysis was conducted using standard methods.

In line with the recommendations implemented by the WHO GISRS Expert Committee for Antiviral Resistance in Influenza, virus susceptibility was classified based on fold differences compared with the respective NA inhibitor median IC_50_ of the matching subtype/type.^15^

## Peramivir susceptibility

The peramivir susceptibility of 2548 influenza A and 1238 influenza B viruses was tested, of which 96·8% (580/599) of A(H1N1)pdm09 and 99·4% (1231/1238) of influenza B viruses demonstrated normal peramivir inhibition, while all A(H3N2) isolates (*n* = 1949) exhibited normal peramivir inhibition. The mean (±standard deviation) peramivir IC_50_ of the influenza B viruses with normal inhibition was 0·74 ± 0·33 nm, four-fold higher than the mean IC_50_ of the influenza A(H1N1)pdm09 or A(H3N2) viruses (Table 1). In addition, there was no significant difference in the median peramivir IC_50_s of B Victoria compared with B Yamagata lineage viruses exhibiting normal inhibition.

**Table 1:** Overall median and mean peramivir and laninamivir IC_50_ of influenza viruses with normal inhibition*

NA inhibitors	A(H1N1)pdm09 IC_50_ (nm)	A(H3N2) IC_50_ (nm)	B IC_50_ (nm)
Peramivir			
No. tested, *n*	580	1949	1231
Median (p25, p75)	0·13 (0·10, 0·24)	0·20 (0·15, 0·27)	0·68 (0·50, 0·90)
Mean ± SD	0·17 ± 0·10	0·18 ± 0·08	0·74 ± 0·33
Laninamivir			
No. tested, *n*	511	1950	1238
Median (p25, p75)	0·22 (0·19, 0·30)	0·60 (0·45, 0·80)	2·37 (1·92, 3·00)
Mean ± SD	0·27 ± 0·05	0·62 ± 0·05	3·26 ± 0·26

* To determine the overall median and mean peramivir and laninamivir IC_50_ of influenza viruses with normal inhibition, the median for all viruses tested in 2009, 2010, 2011 and 2012 was initially calculated. Viruses with IC_50_ 10-fold or more (or five-fold or more for influenza B) above the median were then removed from the data set. The final median and mean IC_50_ of normal inhibition viruses were then recalculate and are displayed in Table 1.

**Figure 1:**
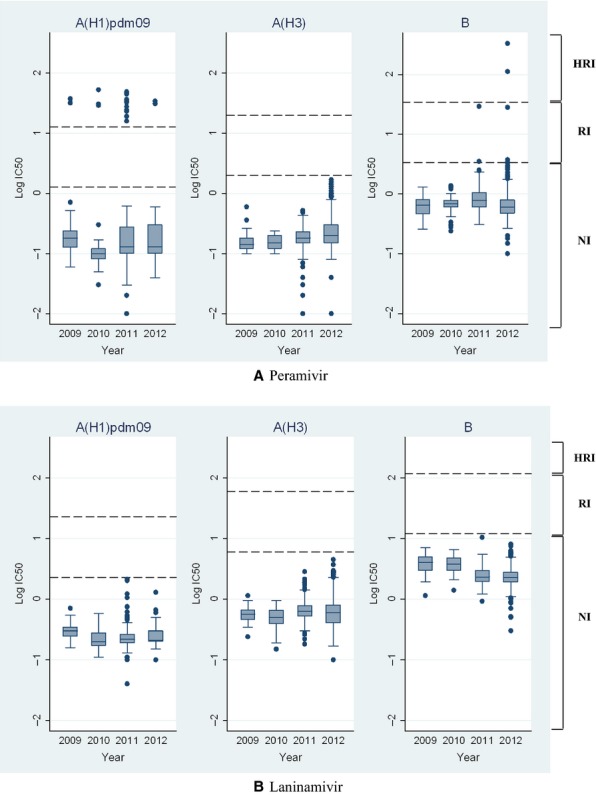
Box-and-whisker plots comparing the distribution of (A) peramivir IC_50_ and (B) laninamivir IC_50_ values (log_10_ transformed) of A(H1N1)pdm09, A(H3N2) and influenza B viruses from 2009 to 2012. The boxes represent the 25th to 75th percentiles, with horizontal lines within each box representing the median IC_50_ values. The whiskers represent the highest and the lowest values situated within the 1·5 IQR plus 75th percentile and the 1·5 IQR minus 25th percentile regions. The dashed lines define the regions “normal inhibition” (NI); “reduced inhibition” (RI); and “highly reduced inhibition” (HRI).

Nineteen A(H1N1)pdm09 viruses (19/599, 3·2%) had highly reduced peramivir inhibition (Figure 1), with a mean IC_50_ value of 31·3 ± 10·3 nm, 241-fold above the median peramivir IC_50_ of A(H1N1)pdm09 viruses with normal inhibition. Genetic analysis of these viruses revealed that they all contained the H275Y NA substitution (N1 numbering, codon 274 in N2 numbering), a mutation known to confer highly reduced oseltamivir inhibition.^12^ Forty-two per cent (8/19) of the H275Y variants detected were from a cluster of cases in Australia in 2011,^16^ but additional strains were also detected in other regions of Australia (2009; 2011; 2012, *n* = 5) and from countries such as Thailand (2010, *n* = 1), Singapore (2010, *n* = 3), Brunei (2011, *n* = 1) and Philippines (2011, *n* = 1) where peramivir and laninamivir are not licensed for use.

**Figure 2:**
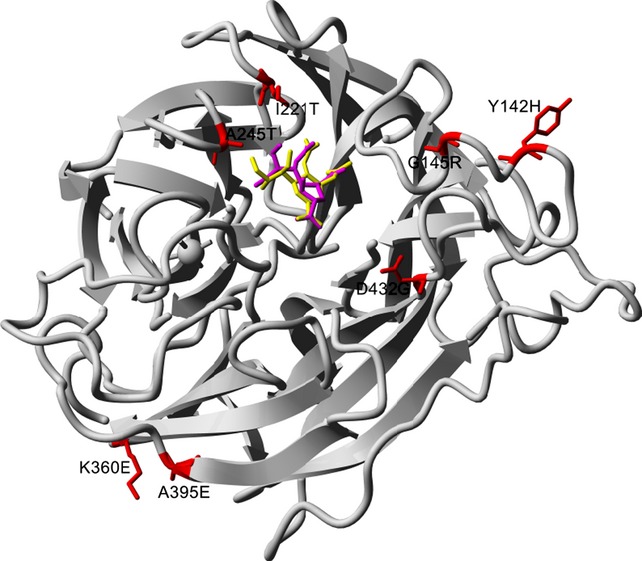
Neuraminidase residue substitutions associated with highly reduced or reduced peramivir susceptibility. The structure is from B/Perth/211/2001 (PDB ID: 3K37, http:\\www.rcsb.org) and visualized with YASARA (http:\\www.yasara.org) Peramivir is represented in magenta and laninamivir (superimposed from PDB ID: 3TIA) in yellow, the mutations are red with wild-type residue shown.

Six influenza B virus isolates were identified as having reduced or highly reduced peramivir inhibition (Figure 1, Table 2). The following influenza B residues are numbered based on straight influenza B NA amino acid numbering starting from the first methionine residue, GISAID accession numbers for sequences of the variant viruses are listed in Table 2. B/Malaysia/210/2012 contained two novel NA mutations Y142H and G145R, with the resulting isolate demonstrating a 487-fold increase in peramivir IC_50_ (Table 2). Y142H is located on the surface of the NA active site and could indirectly affect the binding pocket scaffold loop region including G145R (Figure 2). This may explain how G145R together with Y142H have a strong additive inhibitory effect. Other novel substitutions located in a framework residue (D432G) and outside the active site (K360E and A395E) (Figure 2) were also identified in three influenza B viruses from Thailand and Malaysia with reduced or highly reduced inhibition. B/Bangkok/29/2012, which contained A395E, had a minor five-fold increase in peramivir IC_50_, while B/Malaysia/283/2012 and B/Malaysia/221/2012, which contained K360E and D432G NA mutations, respectively, had 165- and 41-fold increases in peramivir IC_50_ (Table 2). All five of these B variants had normal laninamivir, oseltamivir and zanamivir inhibition, apart from B/Bangkok/29/2012 (A395E NA mutation) which had a five-fold increase in oseltamivir IC_50_. The final two B strains with reduced or highly reduced peramivir inhibition, B/Waikato/21/2011 and B/Wellington/39/2011, have previously been reported to have reduced inhibition to zanamivir and/or oseltamivir.^17^ B/Waikato/21/2011 contained an A245T NA mutation and demonstrated a five-fold increase in peramivir IC_50_, while B/Wellington/39/2011 contained an I221T mutation which resulted in a 43-fold increase in peramivir IC_50_ (Table 2). Variant viruses with either an I221T or I221V NA mutation have also been reported in a number of B viruses from USA and China.^18^,^19^ Compared with wild-type viruses, the I221T variant reported here had a much greater increase in peramivir IC_50_ (43-fold), than reported for the I221V variants from the USA, which exhibited an eight-fold increase.^19^ I221T and A245T are both located near the substrate binding site of the NA (Figure 2). Apart from reductions in peramivir sensitivity, the I221T B variant also demonstrated reduced oseltamivir inhibition^17^, while the A245T mutation was found to affect sensitivity to three of the four NA inhibitors, oseltamivir (20-fold reduction), zanamivir (32-fold reduction) and peramivir (five-fold reduction), even though the residue is not located within the NA active site. The original clinical specimens of many of these isolates were not available to the WHO Collaborating Centre for Reference and Research on Influenza, Melbourne, for sequence analysis (details listed in Table 2) as clinical specimens are often discarded by submitting laboratories once virus isolates are cultured. Therefore, we were unable to investigate whether the mutations had arisen during cell culture, as has been the case for some NAI-resistant variants previously reported.^20^

**Table 2:** Influenza B viruses with reduced or highly reduced peramivir inhibition

Designation	GISAID isolate ID	Lineage	Mutation (s)	Inhibition category	Peramivir IC_50_ (nm)* (fold difference†)	Laninamivir IC_50_ (nm)* (fold difference†)
B/Waikato/21/2011	EPI_ISL_118616	B/Victoria	A245T***	Reduced inhibition	3·48 ± 1·14 (5)	7·30 ± 1·35 (3)
B/Bangkok/29/2012	EPI_ISL_134483	B/Victoria	A395E***	Reduced inhibition	3·73 ± 1·57 (5)	3·81 ± 0·47
B/Malaysia/221/2012	EPI_ISL_122586	B/Victoria	D432G***	Reduced inhibition	28·01 ± 8·75 (41)	1·14 ± 0·33
B/Wellington/39/2011	EPI_ISL_118617	B/Victoria	I221T**	Reduced inhibition	29·35 ± 8·65 (43)	2·53 ± 0·22
B/Malaysia/283/2012	EPI_ISL_128716	B/Victoria	K360E***	Highly reduced inhibition	112·09 ± 37·94 (165)	1·97 ± 0·57
B/Malaysia/210/2012	EPI_ISL_128715	B/Victoria	G145R***; Y142H***	Highly reduced inhibition	331·37 ± 262·20 (487)	3·26 ± 1·06

* Mean IC_50_ ± SD (nm), each virus tested in three independent assays.

** Detected in clinical specimen and virus isolate.

*** Clinical specimen not available.

† Fold difference based on comparison with the influenza B median IC_50_ of viruses with normal inhibition as displayed in Table 1 (only fold difference >3 are listed).

## Laninamivir susceptibility

All 511 A(H1N1)pdm09, 1950 A(H3N2) viruses and 1238 influenza B had IC_50_ values that fell within the normal laninamivir inhibition range. The mean (±standard deviation) laninamivir IC_50_ values for A(H1N1)pdm09 and A(H3N2) viruses were 0·27 ± 0·05 nm and 0·62 ± 0·05 nm, respectively. The mean (±standard deviation) laninamivir IC_50_ for the 1238 influenza B isolates was 3·26 ± 0·26 nm, 5–12-fold higher than the mean IC_50_ of the influenza A(H1N1)pdm09 and A(H3N2) viruses. Again, no difference was observed between the laninamivir susceptibility of the two B lineages.

## Conclusion

Although peramivir and laninamivir are currently only licensed in Japan (and in the case of peramivir, also in South Korea and China), approval in other countries is likely to follow as late-phase clinical trials are completed. Of the viruses analysed, none of the A(H1N1)pdm09, A(H3N2) and influenza B viruses had reduced or highly reduced laninamivir inhibition, while a small number of A(H1N1)pdm09 (3·2%) and B (0·5%) viruses had reduced peramivir susceptibility. Of the viruses considered to have normal inhibition, the mean and median values IC_50_ for peramivir were lower than those for laninamivir for all of the three influenza types/subtypes tested. The clinical implications of these differences, and for the H275Y variant viruses with highly reduced peramivir inhibition, are currently unknown and therefore require further study. However, given that the high concentration of peramivir achieved in the blood following intravenous administration^21^_,_ it could be expected that the H275Y and influenza B variants detected here would be inhibited. However, clinical studies have suggested that peramivir is less effective when treating H275Y variant viruses compared with viruses with normal inhibition.^22^

This study has found no evidence of widespread emergence of viruses with highly reduced peramivir or laninamivir inhibition since the market launch in 2010, although the increased number of community cases of A(H1N1)pdm09 viruses with H275Y NA mutation in 2011 (which had highly reduced peramivir inhibition) was concerning. However, a limitation of this study is that the majority of the viruses tested were from regions where peramivir or laninamivir has not been approved for use. Therefore, although our data suggest that peramivir- and laninamivir-resistant viruses are not spreading from regions where the drugs are being used, further studies are required to assess the susceptibility of Japanese and South Korean viruses collected from both drug-treated and untreated patients.
